# Detection of Antibiotic-Resistance by MALDI-TOF Mass Spectrometry: An Expanding Area

**DOI:** 10.3389/fcimb.2020.572909

**Published:** 2020-11-11

**Authors:** Walter Florio, Lelio Baldeschi, Cosmeri Rizzato, Arianna Tavanti, Emilia Ghelardi, Antonella Lupetti

**Affiliations:** ^1^Dipartimento di Ricerca Traslazionale e delle Nuove Tecnologie in Medicina e Chirurgia, Università di Pisa, Pisa, Italy; ^2^Department of Ophthalmology, Université Catholique de Louvain, Cliniques Universitaires Saint-Luc, Brussels, Belgium; ^3^Dipartimento di Biologia, Università di Pisa, Pisa, Italy

**Keywords:** antimicrobial susceptibility testing, blood culture, rapid AST, microdroplet growth assay, MBT-ASTRA, MALDI-TOF, antimicrobial resistance

## Abstract

Several MALDI-TOF MS-based methods have been proposed for rapid detection of antimicrobial resistance. The most widely studied methods include assessment of β-lactamase activity by visualizing the hydrolysis of the β-lactam ring, detection of biomarkers responsible for or correlated with drug-resistance/non-susceptibility, and the comparison of proteomic profiles of bacteria incubated with or without antimicrobial drugs. Antimicrobial-resistance to a number of antibiotics belonging to different classes has been successfully tested by MALDI-TOF MS in a variety of clinically relevant bacterial species including members of *Enterobacteriaceae* family, non-fermenting Gram-negative bacteria, Gram-positive cocci, anaerobic bacteria and mycobacteria, opening this field to further clinically important developments. Early detection of drug-resistance by MALDI-TOF MS can be particularly helpful for clinicians to streamline the antibiotic therapy for a better outcome of patients with systemic infection, in all cases where a prompt and effective antibiotic treatment is essential to preserve organ function and/or patient survival.

## Introduction

The application of MALDI-TOF MS technology to clinical diagnostic microbiology has provided a new, accurate and robust tool allowing rapid and reliable microbial identification (Ferreira et al., 2011; Barnini et al., 2015; Tanaka et al., 2017; Florio et al., 2018a).

The widespread of multi-drug-resistant bacterial strains, especially in hospital settings, have generated a pressing need for the development of rapid and reliable methods for antimicrobial susceptibility testing (AST), and the potentials of MALDI-TOF MS to achieve this goal have been explored.

Multi-drug-resistance is a particularly dramatic problem in systemic infections (Palacios-Baena et al., 2017), and infections involving critical districts [e.g., eye and orbit, where timely administration of an effective therapy is fundamental for sparing organ specific functions or patient survival (Tsirouki et al., 2018; Choi et al., 2019)].

Therefore, a number of studies investigated the possibility to apply MALDI-TOF MS technology to rapid detection of antibiotic-resistance in bacterial pathogens isolated from bloodstream infections as well as to the detection of antimicrobial-resistance in pathogenic fungi (Florio et al., 2018b).

The present review provides (i) a synthetic, updated overview of the different proposed methods based on MALDI-TOF MS, mainly focusing on its most promising applications and (ii) rapid and accurate information regarding antimicrobial-resistance of clinically relevant bacteria.

## Assessment of β-Lactamase Activity by MALDI-TOF MS

**Figure 1:**
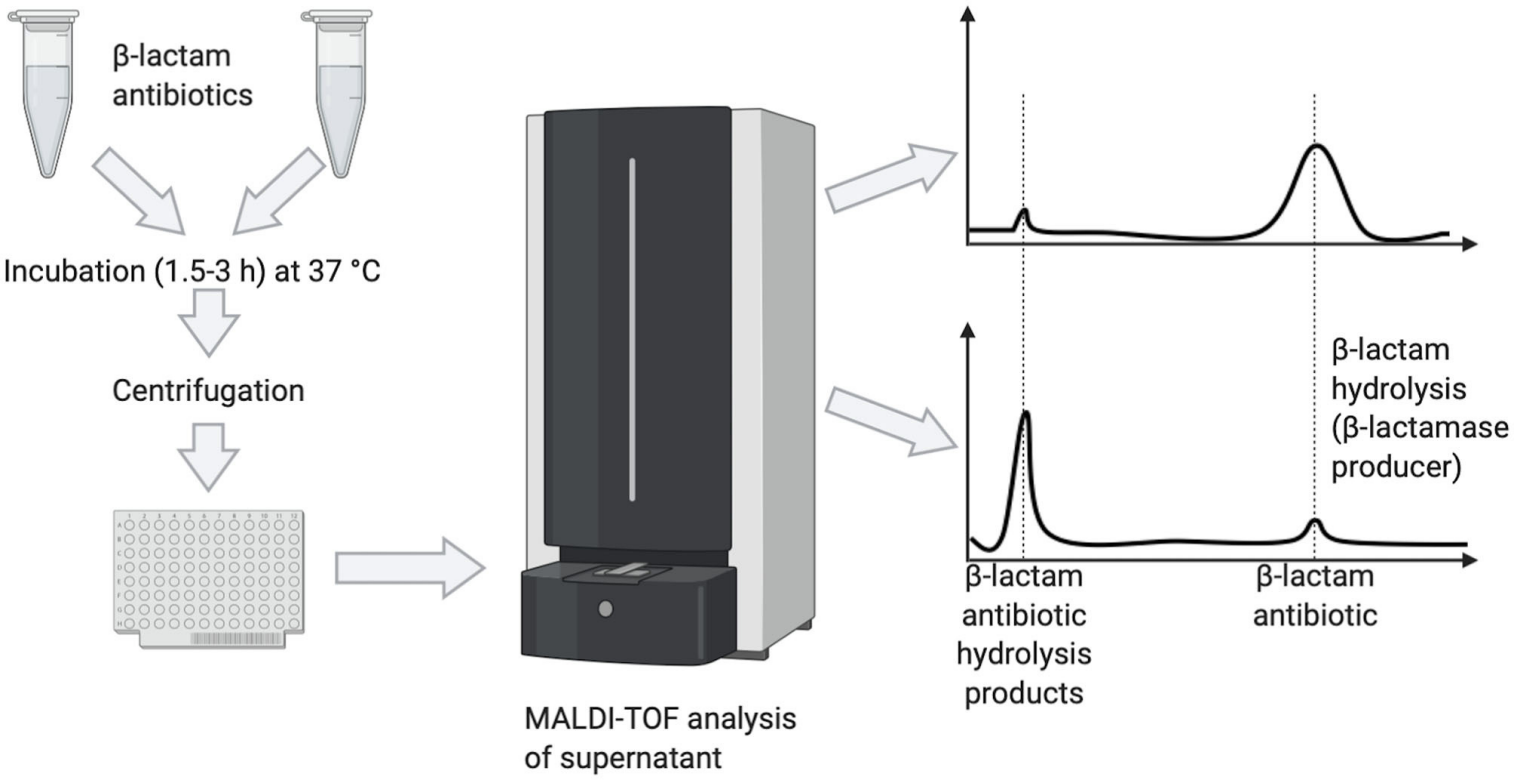
Detection of β-lactamase producers by MALDI-TOF MS based on the hydrolysis of the target β-lactam antibiotic, as visualized by peak disappearance.

One of the first successful applications of MALDI-TOF MS to the detection of antibiotic-resistance (Figure 1) resulted from the observation that the hydrolysis of the β-lactam ring after exposure of β-lactam antibiotics to β-lactamase producing (aerobic and anaerobic) bacteria could be revealed in mass spectra by a decrease of the peak corresponding to the antibiotic and appearance of peaks representing its hydrolysis products (Burckhardt and Zimmermann, 2011; Hrabak et al., 2011; Johansson et al., 2014a,b; Jung et al., 2014b).

A protocol for detection of carbapenemase production by MALDI-TOF MS was developed in *Bacteroides fragilis* strains harboring the *cfiA* gene encoding for the carbapenemase (Johansson et al., 2014b). Hydrolysis of ertapenem was detected within 2.5 h only in *cfiA*-positive strains. The method was successfully applied to screen for carbapenemase activity directly from blood culture bottles inoculated with human blood and spiked with *B. fragilis* strains showing various ertapenem MICs (Johansson et al., 2014a), yielding the results in 3 h. Of interest, the logRQ values of spectra calculated by the software correlated with the MICs for positive strains.

To reduce time for detection of carbapenem-resistance in Gram-negative bacteria causing bloodstream infections, a MALDI-TOF MS-based assay was established by measuring the hydrolysis of imipenem in blood cultures (BCs) spiked with *Pseudomonas aeruginosa, Acinetobacter baumannii*, or *Enterobacteriaceae* producing different carbapenemases (Oviano et al., 2016). The analysis was performed using an MBT Compass STAR-BL module software (Bruker Daltonics), automatically providing a result (sensitivity or resistance) based on the degree of hydrolysis of the antibiotic. This assay, requiring a 30 min-incubation of bacteria with the antibiotic, showed 98% sensitivity and 100% specificity, both reaching 100% with a 60 min-incubation. These results have been confirmed in two large bacterial isolates collections in which the presence of carbapanemase genes was performed in accordance to CLSI method and by PCR (Akyar et al., 2019; Oviano et al., 2020). However, β-lactam resistance is detected only when it is mediated by β-lactamases, whereas the other mechanisms of resistance are not revealed, thus negative results should be confirmed by other assays.

## Identification of Biomarkers Associated With Drug-Resistance

One of the proposed MALDI-TOF MS-based approaches to detect bacterial resistance to carbapenems relies on the identification of plasmids bearing *bla*_*KPC*_ carbapenemase genes (Lau et al., 2014). An extended-spectrum class C beta-lactamase from *A. baumannii*, belonging to the *Acinetobacter*-derived cephalosporinases (ADC) family, has been recently identified (m/z~40,279) and evaluated as a possible biomarker for carbapenem-resistance. Among 51 carbapenem-resistant *A. baumannii* strains, 49 showed a signal at 40,279 ± 87 m/z, whereas four out of 15 carbapenem-susceptible strains showed a signal at the same m/z. The sensitivity and specificity were 96 and 73% in comparison to the microdilution imipenem susceptibility testing, which provides MIC determination (Chang et al., 2018).

Using the ClinProtTools analysis software (v3.0; Bruker Daltonics) to investigate possible differences between the protein patterns of KPC-producing and non-KPC-producing *K. pneumoniae* strains, an 11,109-Da peak was detected in the spectra of 30 out of 34 KPC producers, and it was absent in all non-KPC-producing isolates (Gaibani et al., 2016). Previous findings showed that the 11,109-Da peak is a cleavage product of a hypothetical protein named p019 (Lau et al., 2014). However, as p019 polypeptide was absent in a subset of *bla*KPC-harboring plasmids, negative results should be complemented by confirmatory tests.

**Figure 2:**
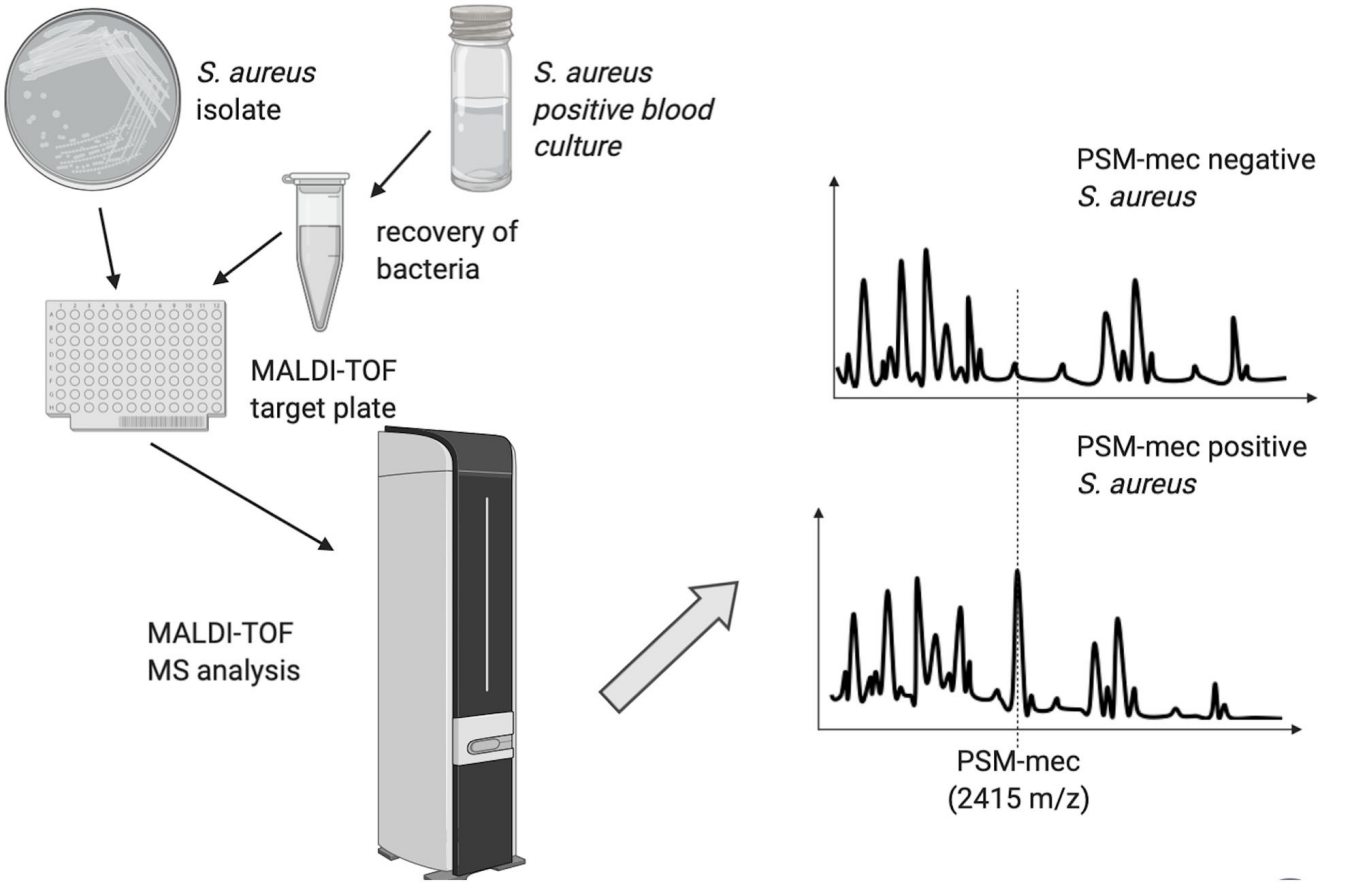
Schematic representation of the MALDI-TOF MS method used to discriminate *Staphylococcus aureus* strains based on the presence of the PSM-mec peak.

The heterogeneous nature of methicillin-resistance in *Staphylococcus aureus* has hindered the possibility to classify methicillin-resistant *S. aureus* (MRSA) and methicillin-sensitive *S. aureus* (MSSA) into two clearly distinguishable groups based on clustering analysis of the spectral profiles of individual isolates (Wang et al., 2013). A partial success was achieved by establishing a method, which relies on the production of a phenol-soluble protein toxin (PSM-mec) by a subset of MRSA strains (Chatterjee et al., 2011), which is detectable by MALDI-TOF MS as a 2415 ± 2.00 m/z peak and may be present in up to half of MRSA isolates (Rhoads et al., 2016; Schuster et al., 2018). The PSM-mec peptide does not cause methicillin-resistance and its biological function is unknown, but its expression is associated with methicillin-resistance. The presence of the 2415 ± 2.00 m/z peak has been shown to predict *mecA* carriage in *S. aureus* with a specificity close to 100%. The software “MBT subtyping module” (Bruker Daltonics), has been developed to detect PSM-mec in the mass spectrum of *S. aureus* isolates, providing an indirect evidence of methicillin-resistance (Figure 2). Although the presence of PSM-mec is associated with methicillin-resistance also in coagulase-negative staphylococci (CoNS), the majority of those does not produce it (Schuster et al., 2018), thus being of limited usefulness to detect methicillin-resistant CoNS.

Identification of possible markers associated with drug-resistance by MALDI-TOF MS has been investigated also in anaerobes (Nagy et al., 2011) [e.g., *cfiA*-positive *B*. *fragilis* can be distinguished from *cfiA*-negative strains by a set of peak shifts in the interval 4,000–5,500 Da, using the MALDI Biotyper software (Bruker Daltonics)]. Two reference spectra were created, one specific for *cfiA*-negative and one for *cfiA*-positive strains. Subsequently, the possibility to screen for carbapenem-resistant *B*. *fragilis* strains directly from positive BCs was demonstrated by comparing ID-spectra of bacilli recovered from spiked BCs with *cfiA*-positive and *cfiA-*negative main spectra (Johansson et al., 2014a).

The increasing prevalence of extensively drug-resistant *P. aeruginosa* infections is due to the global spread of defined high-risk clones. Among them, ST175 is particularly frequent in Spain and France. A MALDI-TOF biomarker peak-based recognition model was evaluated in three collections from Spain and France. Spectra analysis revealed two biomarker-peaks (6,911 and 7,359 m/z) present in all ST175 strains, that most of the susceptible strains lacked. The peak 7,359 m/z was already associated with ST175 (Cabrolier et al., 2015) and the recognition of the second peak increased specificity to 97.8% (Mulet et al., 2019).

With another approach focusing on mass spectrometric analysis of membrane lipids, it was demonstrated that bacterial glycolipids possess species-specific characteristics allowing bacterial identification (Leung et al., 2017). Next, a lipid-extraction protocol was described (Liang et al., 2019) that reduces the identification time to <1 h. This method has been tested to detect antibiotic-resistance and to identify microbes, without requiring culture, using a library of the clinically relevant ESKAPE pathogens and colistin-resistant pathogens expressing the *mcr-1* gene in *trans*. Different biomarkers of resistance were found: *mcr-1*-containing *P. aeruginosa* strains showed a mass shift, 1,446–1,569 m/z, deriving by a PEtN (Δm/z 123) addition to lipid A. The 1,569 m/z ion was not observed in susceptible strains; while *K. pneumoniae* showed a modification of lipid A by AraN (Δm/z 131) attached on the terminal phosphate group of hexa-acetylated lipid A (1,824 m/z), resulting in a modified structure at 1,955 m/z; similarly mass spectrum from *Morganella morganii* with AraN-modified lipid A ions shifted from 1,796 to 1,927 m/z.

The above described methods are specific for one resistance mechanism, thus requiring confirmation of negative results, and have the advantage to use the routinely identification procedure without incubation with the antibiotic, followed by a different spectral analysis, except for the method of Leung, which does not require culture.

## MALDI Biotyper-Antibiotic Susceptibility Test Rapid Assay (MBT-ASTRA)

The MBT-ASTRA is a rapid method for detection of antibiotic-resistance based on a MALDI-TOF MS software tool that calculates and compares the area under the curves (AUCs) of spectra of bacteria either exposed or not to an antibiotic (Lange et al., 2014; Sparbier et al., 2016). The AUCs of the tested bacterial strain are compared to assess bacterial growth. If the microbial strain is susceptible, the AUC of the bacterial suspension with the antibiotic will be reduced compared to that without antibiotic, whereas with a resistant strain the AUCs with or without antibiotic will be comparable.

In principle, the MBT-ASTRA could be used for all classes of antibiotics and microbial species (Ceyssens et al., 2017). The ability of the MBT-ASTRA was evaluated to detect antimicrobial-resistance in a number of randomly selected clinical isolates of *Mycobacterium tuberculosis* and non-tuberculous mycobacteria (NTM). *M*. *tuberculosis* strains were tested for susceptibility to rifampin, isoniazid, linezolid, and ethambutol, and NTM to clarithromycin and rifabutin. The MBT-ASTRA measures bacterial biomass changes after addition of antimicrobial drugs. Using this method, the antimicrobial-resistance profiles of *M*. *tuberculosis* and NTM strains were correctly detected. However, the turnaround time was not shortened for *M*. *tuberculosis*, whereas for NTM was one week earlier.

The MBT-ASTRA approach was evaluated (Jung et al., 2014b) for detecting non-susceptibility against gentamicin and ciprofloxacin in BCs spiked with different *Enterobacteriaceae*, as well as against cefotaxime, piperacillin-tazobactam, and ciprofloxacin in monomicrobial BCs from patients with sepsis from Gram-negative bacteria. To detect non-susceptibility with the MBT-ASTRA method, antibiotics were tested at concentrations that were one dilution higher than the EUCAST susceptibility breakpoints. Overall, the results of microbial identification and susceptibility testing were obtained in ~4 h. In experiments with spiked BCs, a clear separation between susceptible and non-susceptible isolates was obtained for gentamicin based on the observed median relative growth values. For ciprofloxacin, and piperacillin-tazobactam, a small number of isolates were misclassified as susceptible by the MBT-ASTRA mostly when MIC values were close to the antibiotic concentration used in the assay.

Overall, these results indicate that the MBT-ASTRA could be a promising approach for rapid AST of Gram-negative bacteria directly recovered from monomicrobial BCs, but also highlight the need to optimize the method, especially for correct classification of isolates with MIC values close to the breakpoint concentrations, and for some antibiotics.

The MBT-ASTRA was evaluated (Sauget et al., 2018) for rapid detection of amoxicillin- and cefotaxime-resistant *E. coli* isolates from positive BCs. An aliquot of the selected BCs was subcultured for 1 h in antibiotic-free liquid medium before recovery of bacteria and incubation with or without antibiotic, followed by analysis of spectra with the MBT-ASTRA software. Overall, the results of this susceptibility test were available within 4 h after BC positivity. Categorical agreement between the MBT ASTRA and the reference method was 97 and 83% for amoxicillin and cefotaxime, respectively. MBT-ASTRA correctly classified 95 and 84% of the amoxicillin- and cefotaxime-susceptible *E. coli* isolates, respectively, thus MBT-ASTRA may vary depending on the tested antibiotic.

The MBT-ASTRA was evaluated for AST of *B. fragilis* (Justesen et al., 2018), showing a clear difference of the relative growth between a susceptible (ATCC 25285) and a resistant (O18) strain exposed to clindamycin, meropenem, or metronidazole. The accuracy of this method should be confirmed on clinical isolates.

Strength and limitation of this method will be discussed below.

## Assays Based on Detection of Peak Shift After Stable Isotope-Labeling (MBT-Resist)

Another proposed approach to determine drug-resistance in bacteria by MALDI-TOF MS is based on the use of non-radioactive isotope-labeled media (Demirev et al., 2013). Bacteria are grown, in parallel, in two different culture media, one containing ^12^C, and the other one ^13^C as carbon component. The mass spectrum of bacteria grown in antibiotic-containing isotope-labeled media is compared with the mass spectrum of the same strain grown in unlabeled media without antibiotic. Resistant bacteria can grow in the presence of the antibiotic incorporating ^13^C in their polypeptides, which causes a shift of peaks to higher m/z in the mass spectrum.

To evaluate the applicability of the method discriminating MSSA from MRSA strains, culture media containing ^13^C-labeled lysine were used to test oxacillin- and cefoxitin-resistant *S. aureus* clinical isolates (Sparbier et al., 2013). Bacteria were incubated for 3 h in three different conditions: ^12^C-containing medium without antibiotic, ^13^C-containing medium without antibiotic, and ^13^C-containing medium with antibiotic. One out of 14 susceptible strains was misclassified as oxacillin-resistant, and three strains were misclassified regarding cefoxitin-susceptibility/resistance.

Next, peak shifts analysis after incubation for 3 h with either ^13^C-^15^N-labeled or unlabeled L-lysine was established to test meropenem-, tobramycin- and ciprofloxacin-resistant *P. aeruginosa* strains (Jung et al., 2014a). To optimize the assay, meropenem was added 30 min before addition of the stable isotope-labeled amino acid to the test tube, allowing the antibiotic to act. The results revealed concordant classification of susceptible and resistant strains with the reference method (Etest). Theoretically, this method as well as the MBT-ASTRA could be applied to all classes of antibiotics and microorganisms. Drawbacks of these two methods are (i) the incubation time required for microbial growth, (ii) the need to optimize experimental conditions for different isolate-antibiotic combinations, (iii) a correlation between an alteration of mass spectra and MIC values. For *Candida albicans*, the minimal profile change concentrations (MPCCs) [i.e., the minimum drug concentration at which an alteration of mass spectra can be detected, has been highly correlated with the MICs (Marinach et al., 2009)]. The main difference between MBT-ASTRA and MBT-Resist is that the latter requires three different growth conditions.

## Antibiotic-Resistance by Direct-on-Target Microdroplet Growth Assay (DOT-MGA)

An innovative method allowed the detection of antimicrobial-resistance/susceptibility of bacteria incubated with breakpoint concentrations of antibiotics directly on the target plate of MALDI-TOF MS (Idelevich et al., 2018a). The authors tested *K. pneumoniae* and *P. aeruginosa* for resistance to meropenem, but any microbial species and antimicrobial agent could be used, independently from the underlying resistance mechanisms. A 6 μl-incubation volume and optimal/minimum 4 and 5 h incubation times were established for *K. pneumoniae* and *P. aeruginosa*, respectively. In these experimental conditions, 100% sensitivity and specificity were reached for both microorganisms, and the rate of valid tests resulted 100% for *K. pneumoniae* and 83.3% for *P. aeruginosa*. Interestingly, the analysis of spectra after incubation with the antibiotic was performed using the MALDI Biotyper 3.1 software, routinely used for microbial identification.

In another study, the direct-on-target microdroplet growth assay was evaluated on positive BCs from blood samples spiked with meropenem-non-susceptible and meropenem-susceptible *Enterobacteriaceae* isolates (Idelevich et al., 2018b). The best performance was obtained by recovering bacteria from positive BCs, and after a 4 h-incubation of microdroplets with or without meropenem at the breakpoint concentration. In these conditions, 96.3% validity, 91.7% sensitivity, and 100% specificity were achieved.

Recently, a screening panel for detection of extended-spectrum β-lactamase (ESBL) and AmpC β-lactamase activity was developed employing the direct-on-target microdroplet growth assay (Correa-Martinez et al., 2019). The panel was validated on 50 clinical isolates including species of the *Enterobacteriaceae, Hafniaceae, Morganellaceae*, and *Yersiniaceae* families with different mechanisms of resistance (ESBL and/or AmpC) to third generation cephalosporins. The synergistic effect between four cephalosporins and ESBL (clavulanic acid) and/or AmpC (cloxacillin) β-lactamase inhibitors was evaluated to detect effective β-lactamase production. Incubation time of bacteria with or without β-lactamase inhibitors and/or antibiotics was optimized at 4 h. Compared to the PCR results, positive percent agreement values for ESBL, AmpC, and ESBL+AmpC resistance were 94.4, 94.4, and 100% and negative percent agreement values 100, 93.7, and 100%, respectively. The accuracy of the direct-on-target microdroplet growth assay resulted comparable to that of broth microdilution assay, with a time saving of about 14 h, and higher than combination disk tests.

Another MALDI-TOF MS-based method requires incubation of bacteria with different concentrations of antibiotic, recovery of microorganisms before MS analysis (Li et al., 2018) using the MALDI Biotyper 3.1 software. To evaluate the applicability of the method, meropenem-susceptible and meropenem-resistant *A. baumannii* clinical isolates were analyzed after a 4 h-incubation with different concentrations of antibiotic. The isolates were classified as resistant if identification was achieved with scores ≥1.7 after incubation with meropenem at the breakpoint concentration, and as susceptible if identification failed (scores <1.7). The authors recommend applying a threshold of 2 μg/mL for drug-resistance to lower the probability of very major errors (false susceptibility) due to insufficient bacterial growth in the presence of the antibiotic.

## Discussion

In this review, recent advances and newly proposed methods for rapid detection of antimicrobial-resistance in bacterial pathogens by MALDI-TOF MS have been summarized and discussed. Timeliness and accuracy of test results are crucial factors for clinicians to decide and promptly administer an effective and targeted antimicrobial therapy, especially in life-threatening infections or when crucial organs and functions, such as sight are at risk. Further research efforts will be made to refine and optimize MALDI-TOF MS-based assays to obtain accurate and reliable results in the shortest possible time. A major focus of future research in this field will be to achieve standardization of methods and simultaneous susceptibility testing of microbes to various classes of antimicrobials, because of the widespread of multi-drug-resistant microorganisms. So far, only two commercially available kits with software for automated interpretation of spectra have been authorized in Europe to detect or carbapenemase activity or resistance toward 3rd generation cephalosporins in clinical microbiology laboratories.

In conclusion, the possibility to detect peaks associated with drug-resistance directly in MALDI-TOF mass spectra of microbial isolates provides early, useful, though limited information that can help clinicians to streamline empirical antimicrobial therapy, as it is the case with some proposed markers for carbapenem-resistance.

The development of new analytical algorithms, automation of procedures, and optimization of assays are expected to expand and refine the clinical applications of MALDI-TOF MS in clinical diagnostic microbiology.

## Author Contributions

WF and AL contributed to the conception and design of the study. WF wrote the first draft of the manuscript. All authors contributed to manuscript revision, read, and approved the submitted version.

## Conflict of Interest

The authors declare that the research was conducted in the absence of any commercial or financial relationships that could be construed as a potential conflict of interest.
